# Isolated Medial Malleolar Fractures: Current Concepts in Management

**DOI:** 10.7759/cureus.75934

**Published:** 2024-12-18

**Authors:** Sunandan Datta, Bratati Bandyopadhyay, Siddharth Khadilkar, Muhammad Tahir, Krishnakumar Subbaraman, Gaji M Hasan, Gourab Bose, Santanu Pattanayak

**Affiliations:** 1 Trauma and Orthopaedics, East Kent Hospitals University NHS Foundation Trust, Margate, GBR; 2 Trauma and Orthopaedics, Aneurin Bevan University Health Board, Newport, GBR; 3 Trauma and Orthopaedics, University Hospitals Birmingham NHS Foundation Trust, Birmingham, GBR; 4 Orthopaedics, East Kent Hospitals University NHS Foundation Trust, Canterbury, GBR; 5 Trauma and Orthopaedics, Queen Elizabeth Hospital Birmingham, Birmingham, GBR; 6 Trauma and Orthopaedics, Prafulla Chandra Sen Government Medical College and Hospital, Arambag, IND; 7 Trauma and Orthopaedics, North West Anglia NHS Foundation Trust, Peterborough, GBR; 8 Orthopaedics, Calcutta National Medical College and Hospital, Kolkata, IND

**Keywords:** american orthopaedic foot and ankle society (aofas), american orthopaedic foot and ankle society (aofas) ankle-hindfoot scores, ankle fracture management, deltoid ligament complex, medial malleolar fractures, trauma

## Abstract

Isolated medial malleolar fractures (IMMFs) are uncommon and often occur with other ankle injuries, complicating their treatment and management. This review aims to compare the complication rates and functional outcomes of surgical versus conservative treatment for IMMFs in skeletally mature patients. The literature suggests that for IMMFs with less than 2 mm of displacement, conservative treatment provides functional outcomes similar to surgical interventions, with minimal complications. However, for fractures with greater displacement, surgical management is typically preferred due to its superior functional outcomes and low complication rates. Additionally, this review explores the biomechanics of various surgical constructs used to manage IMMFs. It provides insight into the most effective techniques for achieving stable fixation and promoting optimal recovery. By synthesizing evidence from the current literature, this review contributes to the ongoing debate regarding the management of IMMFs, emphasizing the importance of individualized treatment plans based on fracture characteristics and patient factors.

## Introduction and background

Isolated medial malleolar fractures (IMMFs) are relatively rare, and the literature on their treatment is both limited and contradictory. These fractures frequently occur alongside other types of ankle injuries, adding to their complexity [1]. IMMFs account for approximately 7.6-9.4% of all ankle fractures [2,3].

Traditionally, IMMFs were treated surgically due to their propensity for painful non-unions [4]. However, recent studies have advocated for considering conservative management for all IMMFs [5]. Some guidelines have taken a more nuanced approach, recommending conservative treatment specifically for fractures that are non-displaced or have minimal displacement (≤2 mm) [6]. This approach is based on the observation that these types of fractures can heal well without surgical intervention, offering similar functional outcomes without the added surgical risks. Consequently, the debate between surgical and conservative treatment approaches continues.

This review article aims to delve into this ongoing debate by comparing the complication rates and functional outcomes of surgically versus conservatively treated IMMFs in skeletally mature patients. By examining the latest evidence and clinical practices, we seek to provide a clearer understanding of the optimal treatment strategies for these challenging fractures. 

## Review

Anatomy and biomechanics

The medial malleolus is the distal medial process of the tibia, which articulates with the medial facet of the talus. It includes the anterior and posterior colliculi, which serve as attachment points for the deltoid ligament [7]. This ligament, composed of superficial and deep parts, is crucial for ankle stability. The superficial part resists hindfoot eversion, while the deep part prevents external rotation of the talus [8]. Additionally, the superficial deltoid ligament stabilizes the talonavicular and subtalar joints [9]. 

**Figure 1:**
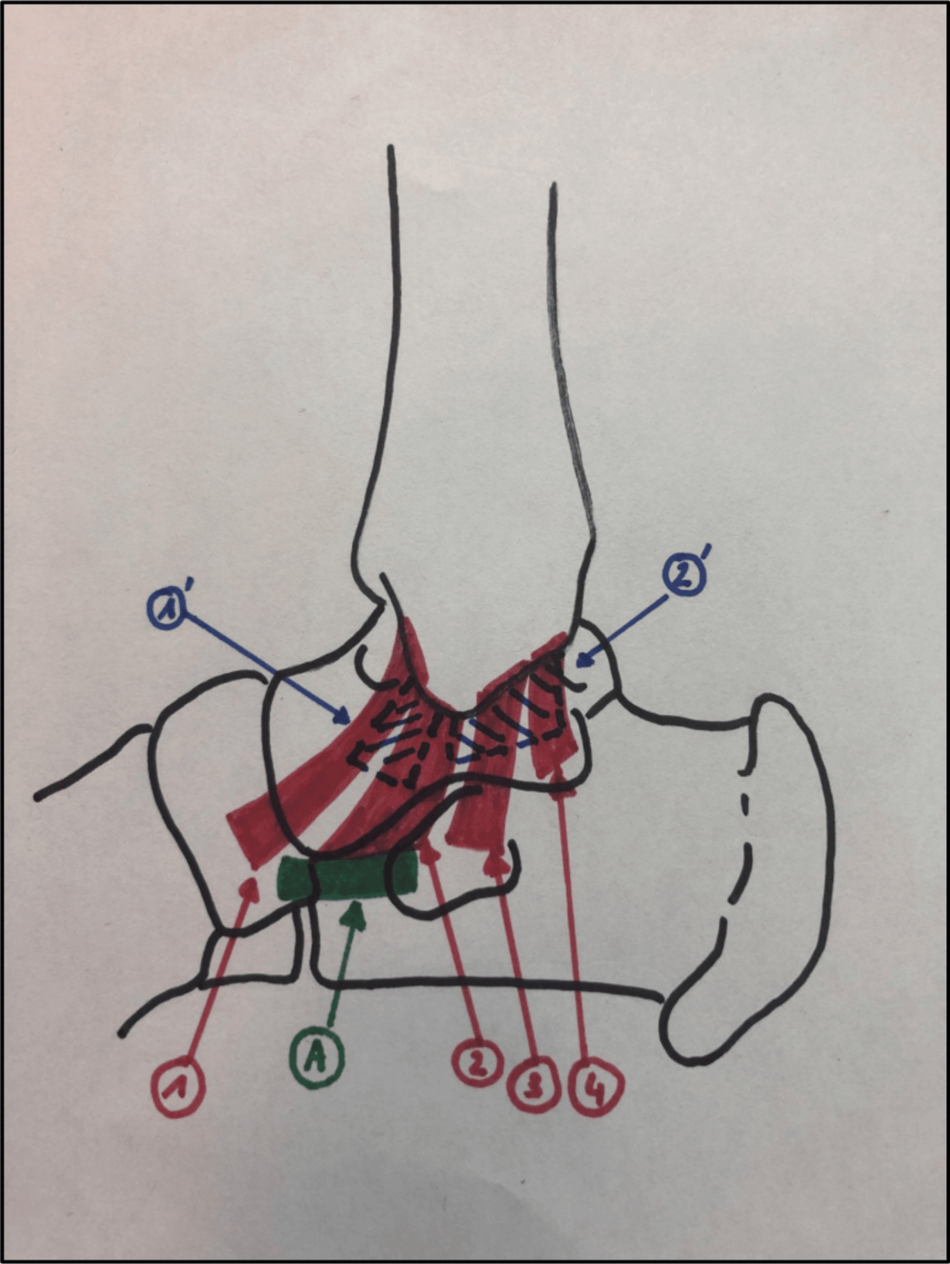
Deltoid ligament complex Superficial bundle, comprising the tibio-navicular ligament (1), tibio-spring ligament (2), tibio-calcaneal ligament (3), and posterior tibio-talar ligament (4). Deep bundle, comprising the anterior (1′) and posterior (2′) tibio-talar ligaments. A: spring ligament. Image Credit: Colin et al. [13]. Reprinted with permission from Orthopaedics & Traumatology: Surgery & Research through CCC RightsLink (License ID: 1550964-1)

The tibiotalar joint endures significant stress, which can be up to three to four times the body weight [10]. The deltoid ligament, also known as the medial collateral ligament (MCL), stabilizes the ankle under eversion, valgus, and rotational stress. The deep component provides stability by restricting lateral translation, abduction of the talus, and eversion of the ankle. The superficial bundle protects against external rotation and valgus stress in the foot and hindfoot [11,12]. Figure 1 illustrates the deltoid ligament complex in a simplified diagram [13].

Both the deltoid ligament and the medial malleolus prevent the medial dislocation of the talus in a dorsiflexed ankle. In a plantarflexed ankle, the deep deltoid ligament internally rotates the talus and prevents its medial subluxation [14].

This complex anatomy underscores the importance of medial structures in maintaining ankle stability. A fracture of the medial malleolus disrupts these stabilizers. Articular incongruity leads to decreased joint contact area and increased contact stresses and peak pressures [15], resulting in ankle joint instability and subluxation.

According to the Lauge-Hansen classification, which is based on injury mechanisms, IMMFs with a transverse fracture line can result from supination-external rotation-, pronation-external rotation-, and pronation-abduction-type injuries. Vertical medial malleolar fractures are usually caused by supination-adduction injuries [16].

Radiography and classification

A medial malleolar fracture is most clearly seen on an anteroposterior radiograph of the ankle, which provides a detailed view of the fracture's location and alignment. For more complex fractures that might extend into the tibial plafond, a computed tomography (CT) scan can be performed. CT scans offer a more comprehensive evaluation, allowing for the detection of subtle fracture lines and a better understanding of the fracture's extent, which is crucial for planning effective treatment.

**Figure 2:**
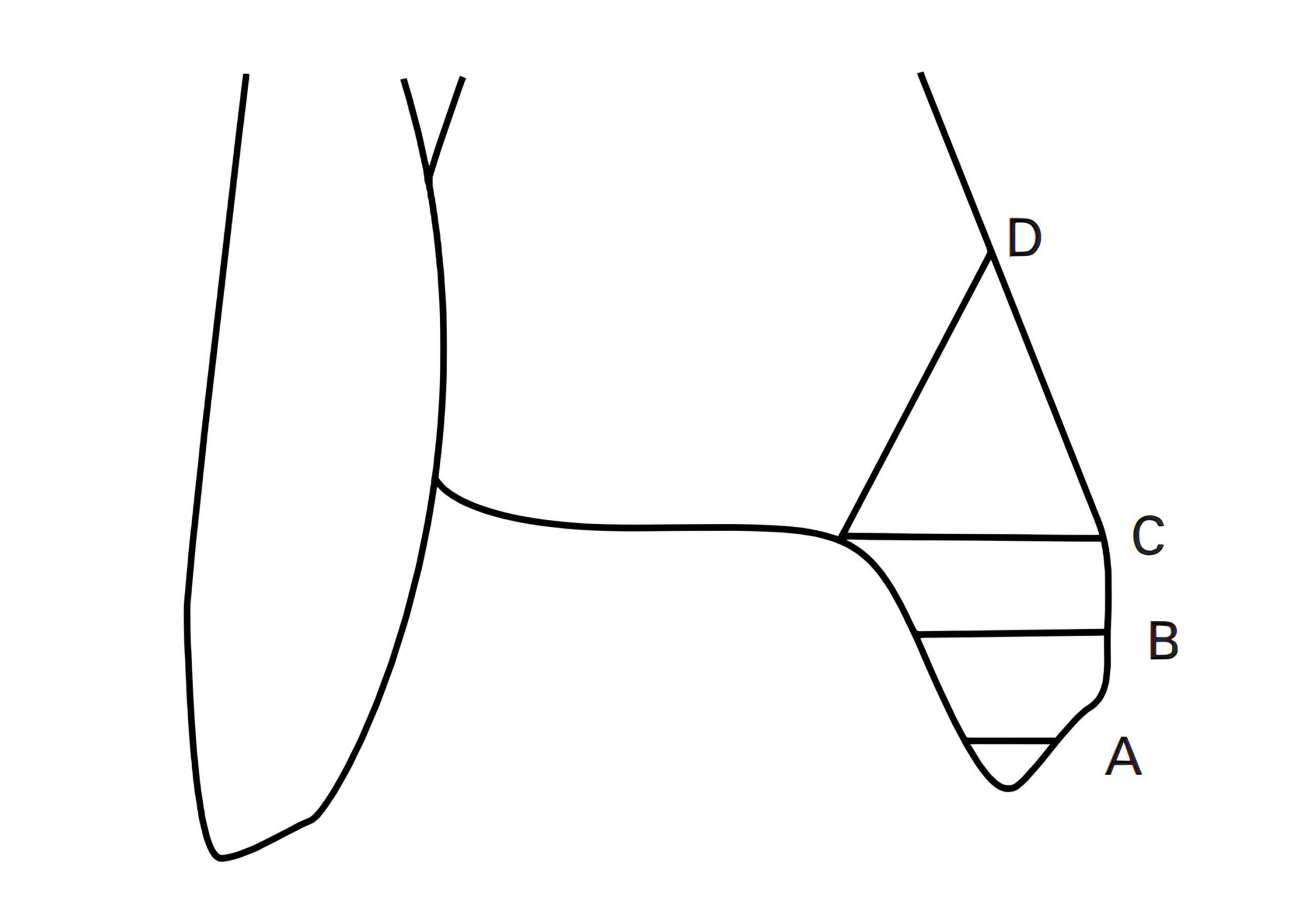
Anatomical classification for isolated medial malleolar fractures (A) Avulsion fracture of the tip of the malleolus. (B) Fracture occurring between the tip of the malleolus and the level of the plafond. (C) Fracture at the level of the plafond. (D) Fracture extending vertically above the level of the plafond. Image Credit: Herscovici et al. [5]. Reprinted with permission from the Journal of Bone and Joint Surgery (British Volume) through CCC RightsLink (License ID: 1549954-1)

Herscovici et al. developed an anatomical fracture classification that helps clinicians systematically categorize IMMFs [5]. Table 1 and Figure 2 illustrate this classification.

**Table 1:** Herscovici classification for isolated medial malleolar fractures Table Credit: Herscovici et al. [5]

Type	Description
A	Avulsion fracture of the tip of the malleolus.
B	Fracture occurring between the tip of the malleolus and the level of the plafond.
C	Fracture at the level of the plafond.
D	Fracture extending vertically above the level of the plafond.

Principles of treatment

The primary objective of fracture treatment is to restore articular congruity and ensure a stable, pain-free ankle, allowing for early patient mobilization. While these fractures were previously routinely fixed to ensure anatomical reduction and stable fixation, treatment strategies have evolved over time.

Determining the appropriate treatment method is crucial. A 2017 retrospective study by Hanhisuanto et al., involving 137 patients, concluded that IMMFs with less than 2 mm of displacement do not require surgery. For fractures with more than 2 mm of displacement, surgical fixation is shown to have superior outcomes compared to nonoperative management [6,17].

All patients should undergo standard anteroposterior, mortise, and lateral radiographs of the ankle following a thorough clinical examination to rule out concomitant injuries. Associated proximal fibular fractures, indicating Maisonneuve injury, should be ruled out through clinical examination and radiographs. Patients with suspected ankle instability should be evaluated using the manual external rotation stress test under fluoroscopy [18].

Patients with IMMFs and ankle instability require surgical fixation to stabilize the ankle joint, regardless of fracture size and displacement. However, as noted by Herscovici et al., these fractures are not typically associated with ankle instability, which is more characteristic of bimalleolar and trimalleolar fractures [5].

Conservative management involves using a non-weight-bearing below-knee plaster cast for approximately six weeks, with the foot in slight inversion. Patients should be monitored with outpatient radiographs at two, four, and six weeks to check for fracture displacement and mortise positioning. After six weeks, patients can weight-bear as tolerated in walking boots, and physiotherapy should commence to prevent ankle stiffness.

Operative management is reserved for Herscovici type B and C fractures with displacement greater than 2 mm. Type D fractures require anti-glide plate stabilization as they extend into the tibial plafond [5]. Various surgical techniques exist, with some authors preferring one or two 3.5 mm partially threaded cancellous lag screws (35-45 mm length) inserted at 90 degrees to the fracture following satisfactory open reduction [19,20]. A randomized controlled trial by Afifi et al. recommended using both partially threaded and fully threaded screws, as both showed comparable clinical and radiological outcomes [21].

Other techniques may include tension band wire constructs and Kirschner wires [22]. Newer methods advocate using ankle arthroscopy to aid in fracture fixation, which has shown lower postoperative pain, as observed by Liu et al. [23].

Functional outcomes 

For most minimally displaced fractures, conservative management is the treatment of choice. After a period of immobilization and non-weight-bearing, followed by gradual weight-bearing, most patients fully recover ankle function. In contrast, surgical intervention carries risks such as infection, hardware irritation, or improper bone healing, which could affect long-term outcomes. However, surgery allows for accurate anatomical reduction, essential for maintaining articular congruity [20,22].

Patient-reported outcome measures (PROMs), like the Olerud-Molander Score (OMS) and the Foot and Ankle Outcome Score (FAOS), are effective indicators of functional outcomes for these patients. The Visual Analog Scale (VAS) is reliable for measuring pain as a secondary outcome. Complications can be assessed by examining the rates of non-union and revision surgery in these fractures.

A 2017 study by Hanhisuanto et al. showed similar OMS, FAOS, and VAS scores for patients undergoing conservative management compared to those undergoing surgery for IMMFs with less than 2 mm displacement [6].

Herscovici et al. treated 57 patients conservatively, with a mean displacement of 3.8 mm. Despite the relatively large displacement, the mean AOFAS score was 89.8, with only two incidents of non-union. IMMFs with a mean displacement of 3.9 mm for type B fractures treated conservatively showed an AOFAS score of 77.2, with no incidents of non-union. For type C fractures, the mean displacement was 4.7 mm, and the AOFAS score was 96.1, with a non-union rate of 6.9%. Type D fractures, with a mean displacement of 2.2 mm, united after conservative management, resulting in a mean AOFAS score of 93.8 [5].

A prospective study by Turhan et al. reported a mean OMS score of 89 in 47 patients fixed with either arthroscopy-assisted fixation or conventional open reduction and internal fixation. Patients with type B, C, and D fractures were selected for surgery [24].

A study by Kulloli et al. demonstrated comparable functional outcome scores for both conservative and surgical treatment groups [25]. The non-union rate was reported to be 1.7% in approximately 1,803 surgically managed patients and 3.5% in 57 conservatively treated patients, according to a systematic review by Lokerman et al. [26]. The same article reported a revision surgery rate of 0.7% in surgically treated patients and 1.8% in conservatively managed patients. However, comparing the two groups is difficult, as the overwhelming majority of patients in the study underwent surgical treatment.

Patients typically regain range of motion, strength, and stability in the ankle joint, with many returning to their previous activity levels. However, some may experience mild residual stiffness or occasional discomfort, especially with high-impact activities. Conservative management is often successful in preventing long-term complications, though close monitoring is essential to detect potential issues such as malunion or delayed healing [5].

In contrast, surgical intervention, required for more complex, displaced fractures or instability, also generally yields positive functional outcomes, though recovery tends to be more protracted. Surgical fixation, typically using plates and screws, stabilizes the fracture and accelerates the healing process, potentially leading to a quicker return to normal activities compared to conservative methods. However, surgery carries risks such as infection, hardware irritation, or improper bone healing, which could affect long-term outcomes. Rehabilitation is crucial in optimizing functional recovery for both treatment approaches, and patients who follow prescribed exercises and return to activity gradually tend to experience better outcomes [20,21]. Overall, most individuals with IMMFs achieve good long-term functional results, with a high percentage regaining full or near-full ankle function, regardless of the treatment approach [17].

Discussion

Our review highlights that the existing literature provides clear guidance on managing IMMFs with less than 2 mm displacement. The preferred treatment for these fractures is conservative management, as functional outcomes are comparable for both nonoperative and surgical approaches, according to the systematic review by Lokerman et al. [26].

Although patients undergoing conservative management need to remain non-weight-bearing for six weeks, this approach ensures predictable clinical outcomes with minimal complications. Effective physiotherapy and rehabilitation post-injury often help patients overcome any residual stiffness and discomfort [27].

The degree of fracture displacement determines the treatment strategy, aiming to maintain articular congruity essential for medial ankle stability [6].

Two of the most reliable studies in this area show excellent clinical outcomes and no cases of non-union in patients managed conservatively for IMMFs with ≤2 mm displacement [5,6].

For fractures with greater displacement, surgical treatment has demonstrated excellent functional outcomes and low complication rates [6]. Traditional fixation methods, including fully or partially threaded lag screws, tension band wiring, single or double screws, and arthroscopy-guided fixation, have shown comparable clinical and radiological outcomes [20-24].

A study by Pollard et al. involving 44 patients demonstrated that fully threaded bicortical 3.5 mm screws and partially threaded cancellous lag screws produced similar outcomes in terms of union, PROMs, and complication rates [20]. However, biomechanical tests showed that fully threaded bicortical screws had superior pull-out strength and greater compression at the fracture site. This advantage is particularly significant in short oblique medial malleolar fractures under transverse and tension loading conditions. Fowler et al.'s study concluded that bicortical fully threaded screws provided the stiffest construct, followed by unicortical partially threaded cancellous screws and tension band wiring. The tension band wiring with a fiber-wire construct was found to be the least mechanically stable option [28].

The study by Herscovici et al. indicates that fractures with displacement greater than 2 mm do not always require surgery; conservative management remains a viable option. However, the sample size and follow-up duration of this study are insufficient to establish a consensus on the optimal treatment for fractures with >2 mm displacement [5].

A randomized clinical trial by Carter et al. in 2024 has shown that the OMS difference between non-fixation and fixation groups at one year was 7.5. This was not found to be statistically significant. Their study also observed a radiographic non-union rate of 20% in the non-fixation group. However, this was not clinically relevant as most of these patients were asymptomatic. It is to be noted that this study did not focus on IMMFs alone and also included patients with bimalleolar and trimalleolar injuries [17].

After a careful study of the available literature on this topic, Lokerman et al.'s recommendation provides a systematic approach towards dealing with these fractures. According to their systematic review, all IMMFs with ≤2 mm fracture displacement should be managed nonoperatively. For fractures with >2 mm displacement, the patient's functional demands should be considered before deciding on treatment. For those with the need for rapid recovery and higher demand, surgery should be the treatment of choice. In the rest, conservative management yields the best outcomes [26].

It is also important to stress that surgical fixation allows for active mobilization and protected early weight-bearing. As noted by Smeeing et al., this leads to an earlier return to work, which translates to greater economic benefits in terms of reduced social security payments and lost wages [29].

Our review has several limitations. Firstly, few articles have studied IMMFs in detail. Our review includes studies on other ankle fractures that also involve medial malleolar fractures as an associated injury. Secondly, only two articles have reported outcomes and complications while considering fracture displacement. This is significant because the degree of displacement is crucial in determining the treatment plan. Thirdly, most studies have not classified the IMMFs, which would have provided a more detailed analysis of these fracture subtypes and their outcomes and complications following both types of treatment. This highlights the need for more studies on this topic.

## Conclusions

IMMFs present a unique challenge due to their rarity and the conflicting literature on their optimal treatment. Historically, IMMFs were treated surgically to avoid painful non-unions. However, recent studies suggest that conservative management can be an effective alternative, particularly for non-displaced fractures or those with minimal displacement. This shift towards conservative treatment highlights the importance of individualized patient care, where the treatment approach is tailored to the specific characteristics of the fracture and the patient's overall health and functional demands. 

The ongoing debate between surgical and conservative treatments highlights the need for more comprehensive studies to provide clearer guidance. Our review compares complication rates and functional outcomes between the two treatment methods, contributing to a better understanding of the most effective strategies for managing IMMFs. Ultimately, the goal is to ensure optimal patient outcomes by leveraging the latest evidence and best clinical practices, whether through surgical or conservative means. By doing so, we hope to offer clearer guidance to clinicians and improve the quality of care for patients with these rare fractures. 
